# Prostate Magnetic Resonance Imaging Analyses, Clinical Parameters, and Preoperative Nomograms in the Prediction of Extraprostatic Extension

**DOI:** 10.3390/clinpract11040091

**Published:** 2021-10-09

**Authors:** Natalia Majchrzak, Piotr Cieśliński, Maciej Głyda, Katarzyna Karmelita-Katulska

**Affiliations:** 1 Transplantology, General Surgery and Urology Department, Poznan District Hospital, Juraszow 7-19, 60-479 Poznan, Poland; pcieslinski@gabinet-urologiczny.pl (P.C.); glydam@wp.pl (M.G.); 2 Hepatobiliary and General Surgery Department, Collegium Medicum in Bydgoszcz, Nicolaus Copernicus University, Sklodowskiej-Curie 9, 85-094 Bydgoszcz, Poland; 3 Department of General Radiology and Neuroradiology, Poznan University of Medical Sciences, Przybyszewskiego 49, 60-355 Poznan, Poland; kkatulska@ump.edu.pl

**Keywords:** prostate cancer, radical prostatectomy, predictive nomogram, preoperative nomogram, MRI, planning surgery

## Abstract

Introduction: Proper planning of laparoscopic radical prostatectomy (RP) in patients with prostate cancer (PCa) is crucial to achieving good oncological results with the possibility of preserving potency and continence. Aim: The aim of this study was to identify the radiological and clinical parameters that can predict the risk of extraprostatic extension (EPE) for a specific site of the prostate. Predictive models and multiparametric magnetic resonance imaging (mpMRI) data from patients qualified for RP were compared. Material and methods: The study included 61 patients who underwent laparoscopic RP. mpMRI preceded transrectal systematic and cognitive fusion biopsy. Martini, Memorial Sloan-Kettering Cancer Center (MSKCC), and Partin Tables nomograms were used to assess the risk of EPE. The area under the curve (AUC) was calculated for the models and compared. Univariate and multivariate logistic regression analyses were used to determine the combination of variables that best predicted EPE risk based on final histopathology. Results: The combination of mpMRI indicating or suspecting EPE (odds ratio (OR) = 7.49 (2.31–24.27), *p* < 0.001) and PSA ≥ 20 ng/mL (OR = 12.06 (1.1–132.15), *p* = 0.04) best predicted the risk of EPE for a specific side of the prostate. For the prediction of ipsilateral EPE risk, the AUC for Martini’s nomogram vs. mpMRI was 0.73 (*p* < 0.001) vs. 0.63 (*p* = 0.005), respectively (*p* = 0.131). The assessment of a non-specific site of EPE by MSKCC vs. Partin Tables showed AUC values of 0.71 (*p* = 0.007) vs. 0.63 (*p* = 0.074), respectively (*p* = 0.211). Conclusions: The combined use of mpMRI, the results of the systematic and targeted biopsy, and prostate-specific antigen baseline can effectively predict ipsilateral EPE (pT3 stage).

## 1. Introduction

Surgical treatment of prostate cancer (PCa) is one of the most effective methods of treatment. Critically, the rate of survival depends on the quality of the treatment given [1,2]. The aim of radical prostatectomy (RP) is to achieve oncological radicality while preserving continence and sexual function. Preserving neurovascular bundles (NVBs) during RP is important for maintaining potency and continence in patients [3,4]. Planning the RP and decisions concerning the preservation of NVBs must be based on the preoperative prognosis of the T3–T4 local stage in order to minimize the risk of positive surgical margins (SM) [5]. It has been demonstrated that in the T3 stage, positive SM increase the risk of biochemical (BCR) and clinical recurrence [6,7].

Proper T-staging, especially distinguishing between T2 and T3 stage, is key in deciding the extent of local treatment. Digital rectal examination (DRE) and transrectal ultrasound (TRUS) are useful only in diagnosing stage T3, which is associated with the presence of large changes in the architecture of the prostate [8]. Local staging based solely on physical examination is unreliable and unclear. Nearly one-third of patients with T3 disease are understaged during rectal examination [9]. Thus, accurate and precise surgical techniques preclude the use of these methods as preoperative T-staging.

Multiparametric MRI (mpMRI) of the prostate, when used to assess local staging, has a low sensitivity and high specificity. Recent studies estimated MRI confirmation of the T3 stage at 61% and 88% for sensitivity and specificity, respectively [10]. For assessing extraprostatic extension (EPE/pT3a), the sensitivity is estimated to be even lower at 57% [10].

On the other hand, various clinical factors such as the PSA baseline, Gleason score (GS) from biopsy result, can determine the local stage of the PCa [11,12]. These clinical parameters are used and combined in “traditional” predictive nomograms for preoperative PCa patient assessment [11,12]. Recent studies have shown that supplementing “traditional” predictive models with mpMRI information significantly improves the diagnostic value of such nomograms [5,9,13,14].

Currently, patients with PCa are qualified for RP based on prostate biopsy (systematic biopsy combined with targeted or targeted only) after previous mpMRI. Therefore, novel nomograms, utilizing mpMRI results, targeted biopsy, and clinical data (e.g.PSA), have been developed [5]. MRI-targeted biopsy in combination with systematic biopsy enables the diagnosis of a greater percentage of clinically significant cancers (csPCa, GS ≥ 3 + 4) and more precisely predicts the risk of stage pT3 in the final histopathology [15,16].

Based on mpMRI data and targeted prostate biopsy, an accurate and reliable nomogram for side-specific EPE can be developed. This allows optimization and personalization of decisions on the extension of RP.

Therefore, the primary aim of this study is to establish what parameters (clinical, radiological, and biopsy data) can best predict the risk of EPE for a specific site of the prostate. The secondary aim was to assess and compare novel models including mpMRI results and biopsy results (the Martini et al. model) and traditional models, based only on clinical parameters and biopsy results (Memorial Sloan-Kettering Cancer Center (MSKCC) and Partin Tables), in predicting the risk of EPE in patients qualified for RP after targeted biopsy combined with systematic biopsy based on mpMRI.

## 2. Material and Methods

The initial studied group consisted of 215 patients. Cancer was confirmed by systematic biopsy combined with MRI-targeted biopsy in 45.6% of patients.

**Figure 1:**
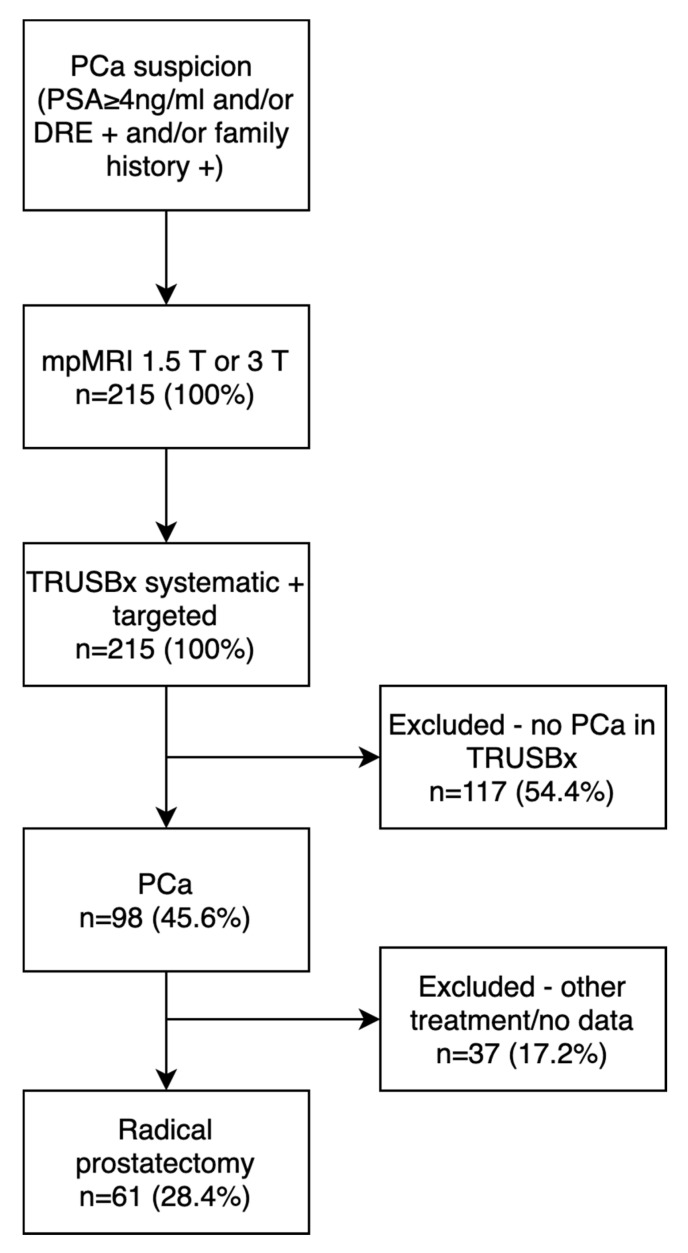
Flow chart of participant inclusion criteria. PCa—prostate cancer; DRE—digital rectal exam; PSA—prostate-specific antigen; mpMRI—multiparametric resonance imaging; TRUSBx—transrectal prostate biopsy.

We retrospectively analyzed 61 patients who met the inclusion criteria of the study (Figure 1).

### 2.1. Imaging

Patients were scanned using a 1.5T (GE Healthcare Medical System Optima MR360, Chicago, IL, USA) or 3T MRI (Siemens HealthCare Magnetom Skyra, Erlangen, Germany). The mpMRI scheme followed the Prostate Imaging—Reporting and Data System v. 2.0 (PIRADS) guidelines of the American College of Radiology (ACR) [17]. It included a multiplanar assessment of the prostate using T1- and T2-weighted imaging, diffusion-weighted imaging (DWI), and dynamic contrast-enhanced (DCE) imaging. Apparent diffusion coefficient (ADC) maps were automatically developed. The mpMRI examinations were evaluated by four radiologists experienced in prostate imaging who knew the PSA levels and DRE results of the patients at the time of the examination. The presence of reported “uncertain EPE” was regarded as positive for EPE.

### 2.2. Prostate Biopsy Technique

Based on the mpMRI findings, transrectal ultrasound-guided systematic and targeted prostate biopsies were performed using a biplane transducer with simultaneous imaging of both planes (BK Medical Flex 400, Herlev, Denmark). Biopsy was performed according to the scheme recommended by the European Association of Urology (EAU): 6–8 cores were taken from each lobe, plus an additional 2–4 specimens from the suspicious lesion depending on its size [18,19,20,21,22]. Each biopsy was performed by one of three urologists, each with at least four years of experience. After PCa diagnosis, patients were qualified for laparoscopic RP.

### 2.3. Surgical Technique

The decision about RP was made on the basis of the EAU progression risk group [22].

Laparoscopic RP was performed either by trans- or extraperitoneal access. The surgery was performed with bilateral, unilateral, or without sparing of NVBs. The NVB sparing technique involved inter- or intrafascial dissection of the bundles, as described by Walz [23,24]. The procedures without NVB preservation involved extrafascial RP [23,24]. The decision to spare NVBs was made depending on the EAU risk group, comorbidities, and the patient’s preferences. Extended lymphadenectomy was performed in the case of high- or intermediate-risk cancer with a predicted probability of lymph node involvement above 7%, according to the Briganti 2017 model [25]. Preoperative risk of EPE was determined according to the mpMRI, MSKCC, Partin Tables, and Martini et al. models [5,11,12]. Each RP was performed by one of three experienced urologists, each with at least four years of experience.

### 2.4. Histopathology

The biopsy material and specimen acquired during RP underwent histopathological examination following the guidelines of the International Society of Urological Pathology (ISUP) 2014 for the pathomorphological diagnosis of PCa [26]. The definition of csPCa was as follows: a Gleason Score ≥ 3 + 4 and/or volume ≥ 0.5 mL and/or extraprostatic extension [17]. All samples from prostate biopsy and RP were evaluated by two genitourinary pathologists who knew clinical and radiological information of the patient.

### 2.5. Statistical Analyses

Median and interquartile ranges (IQR) were calculated for continuous variables, and the frequency and proportion were calculated for categorical variables. The normality of data distribution was assessed using the Shapiro–Wilk test. A Student’s t-test and Mann–Whitney U test with continuity correction were used for continuous variables, while the Chi-square test and Fisher’s exact test were used for nominal variables. Uni- and multivariate logistic regression (UVA, MVA) analyses, taking into account radiological, clinical, and biopsy variables, were performed to determine the factors affecting EPE in the final histopathology. The percentage risk of EPE was assessed using the models of MSKCC, Partin Tables, and Martini et al. [5,11,12]. We used an online calculator from: www.evidencio.com/models, www.mskcc.org/nomograms/prostate/pre_op and www.hopkinsmedicine.org/brady-urology-institute/conditions_and_treatments/prostate_cancer/risk_assessment_tools/partin-tables.html (accessed date 15 March 2021). The expected risk was compared with the final histopathological results. Receiver operating characteristic (ROC) curves were established for the mpMRI examination and each of the nomograms. The sensitivity, specificity, positive predictive value (PPV), negative predictive value (NPV), and area under the curve (AUC) were then calculated. The AUC of the nomograms were compared using the DeLong method. The best cut-off point for a given nomogram was determined using Youden’s index. To evaluate the calibration of the Martini and MSKCC model, Hosmer–Lemeshow (goodness-of-fit) test and calibration plot were performed. A *p*-value < 0.05 was considered statistically significant. Values are reported using the 95% confidence interval (CI).

Considering the maximum size of the lesions described by mpMRI, lesions were divided into two groups: ≥15 mm and <15 mm. This division was based on the PIRADS protocol in which one of the criteria for categorizing lesions is size ≥ 15mm [17]. Continuous variables (% core involvement by PCa) were transformed into nominal variables based on a comparison of the median values between the groups and the values included in the models [5]. Analyses were performed using Medcalc (Mariakerke, Belgium) and SPSS software (IBM, Armonk, New York, NY, USA).

## 3. Results

The characteristics of the study group are presented in Table 1. The median patient age was 66 years. The PSA and PSA density (PSAd) were 6.99 ng/mL and 0.20 ng/mL/mL, respectively. EPE in mpMRI was present in 24.6% of patients. CsPCa (ISUP Grade ≥ 2) was detected during the biopsy in 42.6% of patients.

After RP, 31.2% of patients had an pT3 stage in the RP specimen, and 29.5% had positive SM. Ten patients (52.6%) with EPE had positive SM in the RP specimen. The final histopathology report revealed csPCa in 59% of patients. Seven out of twenty-one (33.3%) patients with spared NVBs had positive SM.

A total of 122 prostate lobes were analyzed. Final histopathology revealed EPE was found in 22.1% of the total prostate lobes in the final histopathology. Two comparative groups were established: the first with diagnosed EPE in a prostate lobe (pEPE+) and the second without EPE in the final histopathological results (pEPE-) (Table 2).

The odds ratio (OR) was determined using the UVA results for each of the factors distinguishing the subgroups. Diagnosis or suspicion of EPE in mpMRI and a maximum lesion size of ≥15 mm were found to be independent predictors of EPE in the final histopathology. In addition, biopsy indicating the presence of csPCa (ISUP Grade ≥ 2) and an extent of PCa in a single core ≥ 50% were the factors that increase the probability of EPE in post-RP specimens (Table 3). There were no differences between the groups in terms of PSA levels as a continuous variable. However, the use of a thresholded PSA level of ≥20 ng/mL significantly predicted the presence of pEPE (Table 3). These results revealed that the combination of mpMRI indicating or suspecting EPE and PSA ≥ 20 ng/mL could significantly determine the risk of EPE for a specific side of the prostate.

**Figure 2:**
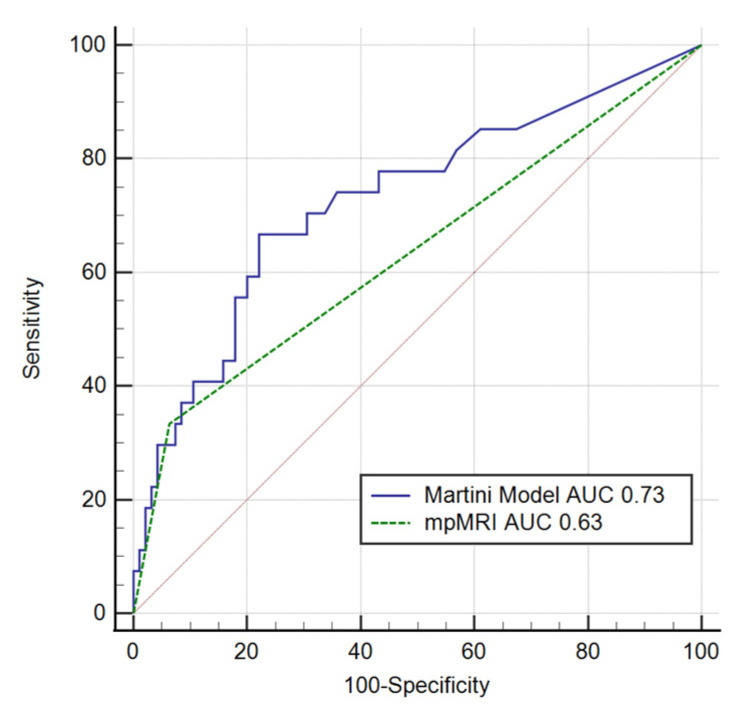
ROCs for the MRI and Martini models for ipsilateral EPE. ROC—receiver operating curve; AUC—area under the curve; mpMRI—multiparametric magnetic resonance imaging; EPE—extraprostatic extension; *p* = 0.131; ΔAUC = 0.09.

**Figure 3:**
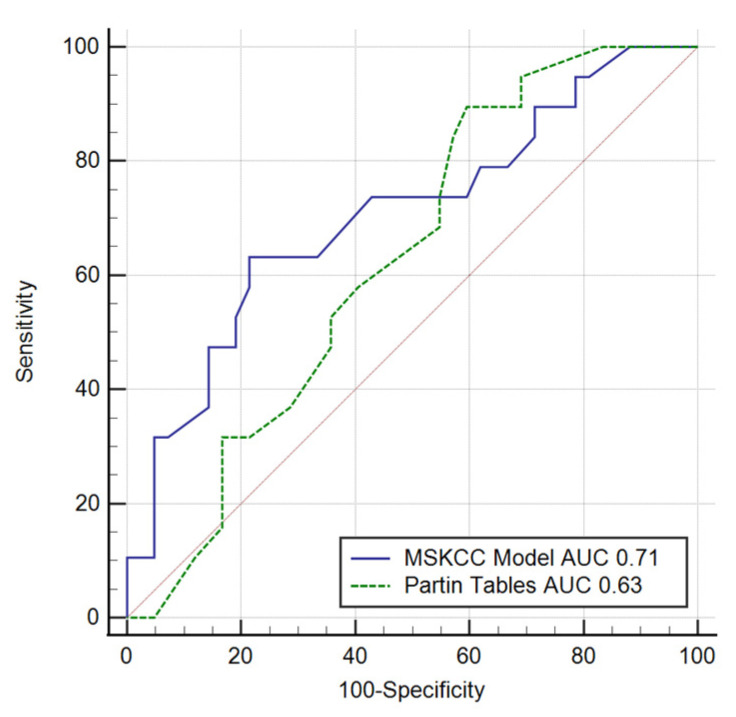
ROCs for MSKCC and Partin Tables for EPE. ROC—receiver operating curve; AUC—area under the curve; MSKCC—Memorial Sloan-Kettering Cancer Center nomogram; EPE—extraprostatic extension; *p* = 0.211; ΔAUC = 0.08.

**Figure 4:**
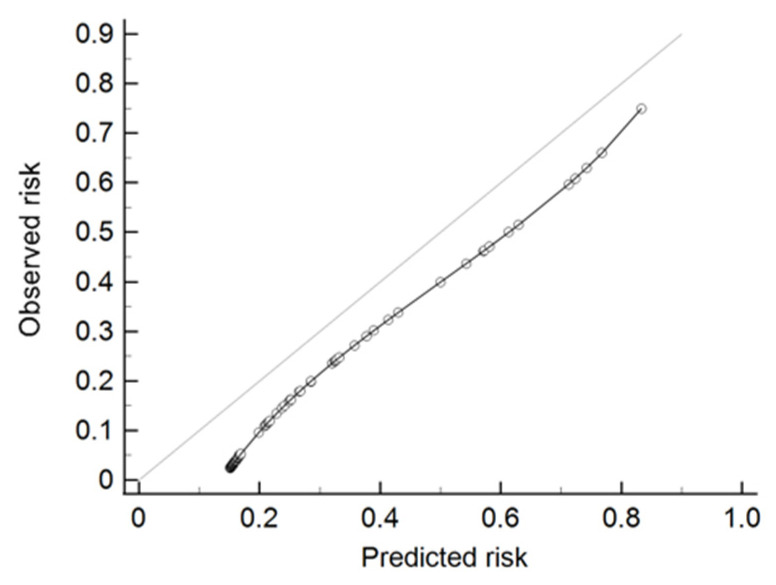
Calibration plot of the Martini nomogram.

The ROC curves for the MSKCC, Partin Tables, and Martini et al. nomograms and mpMRI are presented in Figure 2 and Figure 3. The site-specific model by Martini et al. had the highest sensitivity (66.7%), while mpMRI had the highest specificity (93.7%). The model with the highest AUC for predicting the presence of site-specific EPE was the Martini et al. nomogram (AUC = 0.73, *p* < 0.001); a cut-off point of 5% was proposed. The Hosmer–Lemeshow test for the Martini et al. model confirmed goodness of fit (*p* = 0.71). However, the calibration plot showed an overestimation of the predicted risk (Figure 4).

The AUC for mpMRI examination alone with regard to site-specific EPE assessment was 0.63 (*p* = 0.005). Comparison of the AUC of Martini’s nomogram with that of mpMRI did not show a statistically significant difference (*p* = 0.131, Figure 2)

The AUC for the MSKCC nomogram for EPE prediction was estimated at 0.71 (*p* = 0.007) with a cut-off point of 61%. The Partin Tables nomograms were found to be statistically insignificant, and the AUC was 0.63 (*p* = 0.074). Comparison of the AUC of the MSKCC model with that of the Partin Tables was also not statistically significant (*p* = 0.211, Figure 3). The Hosmer–Lemeshow test for the MSKCC model confirmed goodness of fit (*p* = 0.67). Using calibration curve showed that model underestimated the predicted risk.

The assessed sensitivities, specificities, PPV, and NPV of the models are reported in Table 4.

## 4. Discussion

Good planning of laparoscopic RP needs to take into account the achievement of oncological radicality and good functional results. The choice of the appropriate extension of RP depends primarily on the preoperative prediction of the pT3 stage. NVB sparing surgery in the presence of EPE is associated with an increased risk of positive SM in postoperative specimens [27,28,29,30]. Positive SMs are associated with an increased risk of BCR [31]. However, NVB sparing surgery helps to achieve good functional results, mainly in terms of continence and sexual function, which are crucial for patients’ quality of life [3,4]. Accurate preoperative prediction of the EPE/pT3 stage aids clinical decision making and can minimize the risk of positive SM during RP [31].

In the current study, the analysis of the discriminative value of mpMRI only in terms of stage pT3 assessment indicated a low sensitivity (33%) and high specificity (93.7%) of the examination. The problem of low MRI sensitivity has been repeatedly discussed in the literature. For example, a meta-analysis by de Rooij et al. reported sensitivity and specificity values of MRI for the detection of stage pT3a at 57% and 88%, respectively [10]. Despite its rather poor sensitivity, MRI can enable the selection of the appropriate treatment range [32,33,34]. The urologist’s decision on the extent of RP based on MRI findings has been found to change in 30–50% of cases, with the decision being judged appropriate in 75–90% [32,33,34,35]. Moreover, patients so qualified have a reported lower rate of positive SM (12.4% vs. 24.1% *p* ≤ 0.01) [32,34]. Conversely, the other studies have reported the limitations of MRI in detecting EPE [35,36,37]. MRI alone cannot be used to precisely diagnose EPE [36,37]. The lack of awareness of this limitation can result in wrong surgical decision making and a higher rate of positive SM [36,37]. The aspect of extracapsular micro-infiltration, which is not detected by MRI but is recognized in histopathological examination and classified as stage pT3a, may be important in this respect. Therefore, due to the insufficient predictive value of MRI examination only, it is justified to consider clinical variables in order to achieve a model that can accurately and effectively define the risk of unfavorable histopathology (i.e., pT3).

In the course of this study, it was demonstrated that mpMRI indicating EPE or its suspicion and lesion size of ≥15 mm are independent predictors of pathological EPE for a specific side of the prostate (OR 7.42, *p* < 0.001 and OR 3.67, *p* < 0.001, respectively). Furthermore, the MVA findings in our study demonstrated that mpMRI results indicating EPE or its suspicion with a PSA level of ≥20 ng/mL are the strongest predictors of EPE for a specific side of the prostate.

Subsequently, our comparison of constructed and validated models such as Martini, MSKCC, and Partin Tables nomograms aimed to assess whether the prediction of preoperative risk based on the combination of clinical data and mpMRI is better than prediction based solely on clinical data or imaging examination. AUC analysis indicated that “traditional” models based only on clinical variables, such Partin Tables or MSKCC, have a lower predictive value than models that also take mpMRI data into account [9,13,14,38]. MRI improved the model’s discrimination value [9,13,14,38]. A study by Feng et al. showed that the AUC for the MSKCC and Partin Tables nomograms increased after adding MRI results, changing from 0.86 to 0.92 and from 0.86 to 0.94, respectively [9]. Morlacco et al. demonstrated that Partin Tables with MRI outperformed Partin Tables alone [38]. Similarly, Ryan et al. reported that adding MRI data to the MSKCC nomogram increased the AUC by 0.10 for predicting EPE risk [13]. Additionally, the authors showed that the GS obtained by targeted biopsy, compared to the results of systematic biopsy, significantly improved the discrimination value of the clinical MSKCC model (it increased the AUC by 0.07) [13].

Our analysis of the MSKCC model, which only took clinical data into account, had better sensitivity and AUC than mpMRI alone. However, it should be mentioned that the biopsy results used in the MSKCC nomogram indirectly account for the mpMRI results, as systematic biopsy is performed in combination with targeted biopsy based on mpMRI.

However, we concluded that the nomogram by Martini et al. is the best tool for assessing the risk of cancer extending beyond the prostate, as well as for planning surgical treatment and making decisions to preserve NVBs. The AUC we assessed for the Martini model was 73%, which turned out to be the most sensitive of the compared models and was better than mpMRI alone for estimating EPE. In their own study of the model, Martini et al. estimated an AUC of 82.1% [5]. Although the AUC for the Martini model in the current study was lower than in the original paper, the test of goodness of fit indicated good concordance between observed and predicted risk of EPE with *p* = 0.71. However, the calibration plot showed that the nomogram overpredicted the EPE risk. It should be mentioned that our limited group of patients could have resulted in miscalibration of the model. The other measure of the external validation of the Martini nomogram performed by Soeterik et al. showed good discriminative ability but poor calibration and underestimation of the predicted risk [39].

Martini’s analysis of the number of patients in stage pT3 and with positive SMs in relation to the proposed cut-off point of 20% indicates the highest utility for making decisions to spare NVBs. In the current study, the proposed cut-off point is lower and only differentiates the possibility of EPE or its absence.

Our study has several limitations. This was a preliminary study conducted in one center with a relatively small final group of patients (*n* = 61). We used 1.5T or 3T MRI scanners. A systematic biopsy combined with a targeted biopsy was performed based on mpMRI results. Thus, the biopsy data used in the MSKCC and Partin Tables models differ from the original assumptions of the MSKCC and Partin Tables models [11,12], where only systematic biopsy results are considered. However, despite the above limitations, the conclusions of our study are consistent with the currently available literature on the importance of combining predictive models with mpMRI in the planning of surgical treatment of patients with PCa. We believe that multicenter studies involving larger groups should confirm our conclusions.

## 5. Conclusions

The results of mpMRI indicating the presence of EPE and a high PSA level (above 20 ng/mL) are useful for determining the risk of the pT3 stage in the final histopathology. The use of Martini’s model combining mpMRI and the results of systematic and targeted biopsy, while also taking into account PSA levels, allows surgeons to effectively determine the risk of EPE for a specific side of the prostate in the final histopathological result.

## Figures and Tables

**Table 1:** Characteristics of the study group.

Total *n* = 61	Overall, *n* = 61 (% or IQR)
Age (years)	66 (61.75–69.25)
PSA (ng/mL)	6.99 (5.32–11.0)
PSAd (ng/mL/mL)	0.20 (0.15–0.28)
Prostate volume (mL)	34.0 (29.50–47.20)
DRE	
Abnormal	39 (63.9)
Normal	22 (36.1)
Bx ISUP Grade	
1	35 (57.4)
≥2	26 (42.6)
mpMRI EPE	
Absent	46 (75.4)
Present	15 (24.6)
PIRADS	
5	16 (26.2)
≤4	45 (73.8)
Localization in MRI	
PZ	46 (75.4)
Non-PZ	15 (24.6)
Max diameter of IL in MRI (mm)	13 (9.0–17.0)
RP ISUP Grade	
1	25 (41.0)
≥2	36 (59.0)
EAU Risk Group	
Low	19 (31.1)
Intermediate	38 (62.3)
High	4 (6.6)
pT stage	
2a	9 (14.7)
2b	1 (1.6)
2c	32 (52.5)
3a	14 (23.0)
3b	5 (8.2)
pN stage	
N0	59 (96.7)
N1	2 (3.3)
SM	
Positive	18 (29.5)
Negative	43 (70.5)
Positive in pT2	8 (47.4)
Positive in pT3	10 (52.6)
NVBs	
Yes	21 (34.4)
No	40 (65.6)

Abbreviations: PSA—prostate-specific antigen; DRE—digital rectal exam; Bx—transrectal prostate biopsy; ISUP Grade—Grade International Society of Urological Pathology 2014; mpMRI—multiparametric magnetic resonance imaging; EPE—extraprostatic extension; PIRADS—Prostate Imaging—Reporting and Data System; IL—index lesion; PZ—peripheral zone; non-PZ—zone other than peripheral; RP—radical prostatectomy; SM—surgical margins; NVBs—neurovascular bundle-sparing surgery; EAU—European Association of Urology.

**Table 2:** Analysis of EPE factors for each side of the prostate.

Total *n* = 122 (%, IQR)	pEPE (+) *n* = 27 (22.1)	p EPE (−) *n*= 95 (77.9)	*p*-Value
mpMRI EPE			<0.001
Present	9 (33.3)	6 (6.3)	
Absent	18 (66.7)	89 (93.7)	
PIRADS			0.02
1–2	3 (11.1)	10 (10.5)	
3	3 (11.1)	16 (16.8)	
4	3 (11.1)	24 (25.3)	
5	10 (37.1)	9 (9.5)	
No lesion	8 (29.6)	36 (37.9)	
PIRADS			0.001
≤4	17 (63.0)	86 (90.5)	
5	10 (37.0)	9 (9.5)	
Max lesion diameter in mpMRI			0.005
<15 mm	16 (59.3)	80 (84.2)	
≥15 mm	11 (40.7)	15 (15.8)	
Bx ISUP Grade			0.002
<1	13 (48.1)	75 (78.9)	
≥2	14 (51.9)	20 (21.1)	
% of PCa in bx cores			0.017
<50	13 (48.1)	69 (72.6)	
≥50	14 (51.9)	26 (27.4)	
PSA ng/mL			0.03
<20	24 (88.9)	94 (98.9)	
≥20	3 (11.1)	1 (1.1)	

Abbreviations: PSA—prostate-specific antigen; Bx—transrectal prostate biopsy; ISUP Grade—International Society of Urological Pathology 2014 Grade; mpMRI—multiparametric magnetic resonance imaging; EPE—extraprostatic extension; PIRADS—Prostate Imaging—Reporting and Data System; n—overall number; %—in the brackets.

**Table 3:** Univariate and multivariate logistic regression analyses predicting EPE for each side of the prostate.

	UVA OR (95% CI)	*p*-Value	MVA OR (95% CI)	*p*-Value
PSA	11.75 (1.17–118.04)	0.04	12.06 (1.1–132.15)	0.04
<20 ng/mL–ref.				
≥20 ng/mL				
mpMRI EPE	7.42 (2.35–23.43)	<0.001	7.49 (2.31–24.27)	<0.001
Present				
Absent–ref.				
Diameter of lesion	3.67 (1.42–9.43)	<0.001	NS	
<15 mm–ref.				
≥15 mm				
% of PCa in bx core	2.86 (1.19–6.89)	0.02	NS	
<50–ref.				
≥50				
Bx ISUP Grade	4.04 (1.64–9.95)	0.002	NS	
1–ref.				
≥2				
AUC of	-	-	0.67 (0.57–0.77)	0.008
multivariable				
model (95% CI)				

Abbreviations: UVA—univariate logistic regression; PSA—prostate-specific antigen; Bx—transrectal prostate biopsy; ISUP Grade—International Society of Urological Pathology 2014 Grade; mpMRI—multiparametric magnetic resonance imaging; EPE—extraprostatic extension; PCa—prostate cancer; AUC—area under curve; CI—confidence interval; NS—not significant.

**Table 4:** Assessment of sensitivity, specificity, PPV, NPV, and AUC of the models in terms of EPE.

EPE/pT3	AUC (95% CI)	Sensitivity	Specificity	PPV	NPV	*p*-Value
MSKCC	0.71 (0.57–0.81)	63.2	78.6	57.1	82.5	0.007
Partin Tables	0.63 (0.49–0.75)	89.5	40.5	40	89.5	0.074
Martini et al.	0.73 (0.64–0.80)	66.7	77.9	44.2	89.2	0.001
mpMRI	0.63 (0.54 –0.72)	33.3	93.7	60	83.2	0.005

Abbreviations: EPE—extraprostatic extension; mpMRI—multiparametric magnetic resonance imaging; MSKCC—Memorial Sloan-Kettering Cancer Center nomogram; PPV—positive predictive value; NPV—negative predictive value; CI—confidence interval.

## Data Availability

Data are available from the corresponding author upon reasonable request.
